# Response to the COVID-19 Epidemic: The Chinese Experience and Implications for Other Countries

**DOI:** 10.3390/ijerph17072304

**Published:** 2020-03-30

**Authors:** Wei Liu, Xiao-Guang Yue, Paul B. Tchounwou

**Affiliations:** 1 Business School, Qingdao University, Qingdao 266100, China; 2 School of Sciences, European University Cyprus, 1516 Nicosia, Cyprus; x.yue@external.euc.ac.cy; 3 CIICESI, ESTG, Politécnico do Porto, 4610-156 Felgueiras, Portugal; 4 Department of Biology, College of Science, Engineering and Technology, Jackson State University, 1400 Lynch Street, Box 18750, Jackson, MS 39217, USA

**Keywords:** COVID-19, epidemic, China, emergency control measures, public health

## Abstract

The ongoing outbreak of the novel coronavirus disease (COVID-19) that occurred in China is rapidly spreading globally. China’s bond and strict containment measures have been proved (in practice) to significantly reduce the spread of the epidemic. This was obtained through the use of emergency control measures in the epidemic areas and the integration of resources from multiple systems, including business, community, technology, education, and transportation, across the country. In order to better understand how China has managed to reduce the public health and economic impacts of the COVID-19 epidemic, this editorial systematically reviews the specific measures for infection prevention and control of the disease. The best practices for COVID-19 eradication in China provide evidence-based strategies that could be replicated in other countries.

## 1. Introduction

The COVID-19 outbreak is an ongoing epidemic of viral pneumonia, caused by the severe acute respiratory syndrome coronavirus 2 (SARS-CoV-2) virus [1]. Since the earliest known pneumonia cases of unknown origin that were identified in the Wuhan city of China in December 2019, the epidemic has rapidly spread throughout China in just several months, turning into a serious national public health crisis, and patients with the same symptoms have been gradually documented in other countries [2,3]. Under the circumstance of the significant public health risk that the international spread of epidemic poses to the world, the World Health Organization (WHO) has declared a public health emergency of international concern over the global outbreak of COVID-19 on 30 January 2020, and has escalated it to a global pandemic on 11 March [4]. 

**Figure 1:**
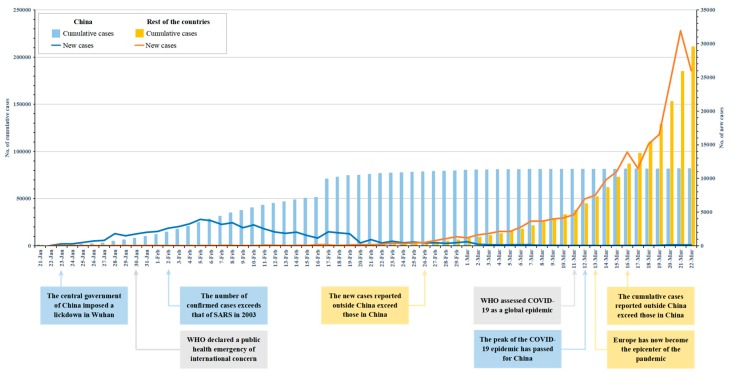
New and cumulative confirmed cases of COVID-19 for China and the rest of the countries by date of report (Data sources: World Health Organization Situation Reports).

Hitherto, there has been a total of 292,142 laboratory-confirmed cases and 12,784 deaths globally as of 22 March, of which 81,498 cases and 3,267 deaths are in China [5]. Even though China is temporarily the country with the largest number of cases, the increase of new cases is falling and there is evidence that the peak of the COVID-19 epidemic has passed for China [6]. Since 19 March, Wuhan, the city where the outbreak started, has reported no new confirmed or suspected cases for five consecutive days [7]. The epicenter of the outbreak was originally in China and has now been transferred to other countries such as Italy, the United States, Spain, and Germany. The WHO confirmed that “Europe has now become the epicenter of the pandemic, with more reported cases and deaths than the rest of the world” [8]. Figure 1 reports the new and cumulative confirmed cases of COVID-19 data for China and the rest of the countries, by date of report. The figure shows a significant difference in the timing of case growth trends between the two location categories. The Chinese government has taken a series of aggressive measures in a timely manner, which have proved to be effective in alleviating the epidemic to a large extent. Consequently, the objective of this editorial is to review a selection of measures undertaken in response to the COVID-19 epidemic in China, and to provide potential evidence and guidance to other countries.

## 2. Response to the COVID-19 Epidemic in China

Since the severe acute respiratory syndrome (SARS) outbreak in 2003, the Chinese government has formulated and implemented the “Regulations on Preparedness for the Response to Emergent Public Health Hazards”, with the intention of establishing a rapid and effective epidemic emergency response mechanism and improving its response capacity, to reduce the degree of emergency disaster to the utmost extent [9]. Compared with the SARS virus, the SARS-CoV-2 virus of the COVID-19 outbreak has the characteristics of a stronger infectivity and a longer incubation period [2]. Additionally, as the COVID-19 outbreak occurred around the time of annual Chinese Lunar New Year holiday, billions of residents returned to their hometown to celebrate the New Year with their families, as is traditional in the Chinese culture, which greatly increased the personnel mobility and traffic congestion in the short term [10]. Wuhan city, the epicenter of the epidemic, is also the core transfer hub of China’s transportation system, which connects most regions in China. These new changes and characteristics have undoubtedly put forward new requirements and challenges for the government to respond to the COVID-19 outbreak. The Chinese government has taken serious comprehensive and nationwide response measures to fight against the spread of COVID-19 and has achieved positive results on the basis of empirical evidence. Here are some major highlights that the government has adopted, to respond to the COVID-19 epidemic.

### 2.1. The Emergency Control Measures of Epidemic Areas

As the epicenter of the epidemic, Wuhan city first announced a travel quarantine area by the government on 23 January 2020, and the quarantine radius was expanded to all other cities in the Hubei province, encompassing a total population of 45 million, by 30 January [11]. All public transportation in Wuhan city, including bus, subway, and ferry services were suspended, and expressways, airports, and railway stations were temporarily closed. All large gatherings including the New Year celebrations were canceled. As the asymptomatic incubation period of COVID-19 could be up to 14 days, all residents were restricted to stay at home in self-quarantine, in order to prevent the spread of the virus. All public places such as shopping centers, schools, restaurants, and cinemas were closed, and food, medicine and medical supplies were uniformly deployed and distributed to all residents, by the staff of the community committees. In order to alleviate the insufficient medical resources in Wuhan city, two emergency specialty field hospitals, Huoshenshan Hospital and Leishenshan Hospital were approved as the centers for patients with COVID-19, which provided approximately 2,400 beds. The two constructions were completed on 2 February and 6 February, respectively. Since 2 February, large venues such as stadiums and convention centers were transformed into 14 mobile cabin hospitals, for the medical observation and treatment of more than 14,000 suspected cases, mild patients, and close contacts. In addition, the Chinese People’s Liberation Army and other provincial medical systems successively dispatched multiple medical rescue teams of more than 42,600 medical staff, to Wuhan city and the Hubei province, ensuring the adequacy of medical resources in the epidemic area.

### 2.2. Multiple Measures of Other Areas

The COVID-19 outbreak has severely disrupted China’s economy, among which transport limitation and restriction has interrupted the supply chain of enterprises, and personnel quarantine has further slowed down business activities [3]. Under such circumstances, many enterprises, especially small and medium-sized enterprises with weak ability to resist risks, are facing the difficult dilemma of the lack of funds and employees. The government has issued a number of targeted policies to support the survival and development of these enterprises during the outbreak. The financing cost of the enterprises has been reduced, reflecting the fact that banks reduced the loan threshold of enterprises, increased loan amounts, and reduced loan interest rates for the period of the epidemic. Enterprises seriously affected by the epidemic could apply for multiple tax relief, and the government also arranged special funds to provide subsidy support for worst-hit industries, such as aviation and transportation industries. The employees of enterprises that returned to work from other areas have also received strong support from the government and were provided with subsidies for chartered airplanes and buses. Fortunately, we have also witnessed many enterprises actively fulfill corporate social responsibility during the outbreak. These enterprises have donate large sums of money and medical equipment to the epidemic areas, some of which, such as the Chinese National Petroleum Corporation, transformed production lines to produce medical protective equipment including masks, protective clothing, and ventilators. As the world’s largest manufacturer and exporter of medical protective equipment, China’s safeguard measures for enterprises to return to work were related to the progress of global infection prevention and control of epidemic. 

Community-based control measures have become the most important part of residents’ quarantine. Many areas across mainland China have implemented the so-called “grid closed management” on a community-basis. The outdoor restriction measure was enforced whereby only one resident from each household was permitted to go outside of the community every two days, and foreign personnel were strictly prohibited from entering the community. Each community kept only one entrance and exit point open, and checkpoints were set up for community staff to perform the identification and temperature tests of each resident entering and leaving the community. Anyone who went outside had to wear a mask. 

Big data and communication technologies were also used to support control measures. WeChat and AliPay, two widely used mobile apps in China, provided the system of Health Codes, on which all outgoing residents had to register and be assigned a QR color code, displaying green, yellow, or red to indicate their health status. Residents were required to scan the QR code when entering and leaving every public place, hence, the outdoor routes of confirmed cases were traced and their close contacts could also be identified. Although this could fall into the debate of over whether personal privacy and human rights were violated, these measures indeed helped to identify confirmed cases and contacts in an effective and rapid manner, to control the spread of the epidemic in China [12].

As the student group is an important component of personnel mobility, the epidemic has also severely impacted the education system. On 27 January, the Ministry of Education of China required all universities, primary and secondary schools, and kindergartens to suspend and postpone the opening time of the new semester. In order to reduce delays in education progress due to the outbreak, schools used internet technology to carry out innovative teaching practices based on online instead of face-to-face teaching, which also brings a rare development opportunity to online education platforms. Many universities also showed significant initiatives in taking on social responsibilities, opening up education platforms to the society without charge, sharing high-quality course resources, and in these ways, contributed to the infection prevention and control of the epidemic [13,14].

The outbreak has also brought a direct and sustained impact on the transportation system. Since 20 January, the public transport places including airports, railway stations, bus stations in all regions started the temperature tests for passengers when entering and leaving the stations. In order to ensure the transportation of emergency supplies and reduce the cost of raw materials for the enterprise resumption, the Ministry of Transport of China announced that vehicle tolls on nationwide expressways were waived during the period of 17 February to the end of the infection prevention and control of epidemic. Due to the reduced demand and residents’ quarantine, the inter-provincial bus was suspended, and most of the aircraft flights and rail services were temporarily canceled. Since March, as the peak of the epidemic has passed for China and the epidemic has begun to spread on a large scale in other countries, the new focus of the response measures of the transportation system has shifted to a new stage of strictly preventing the epidemic from being imported from abroad. Since 23 March, all international flights must arrive in 12 designated first-entry cities, and all entry personnel much be forced to perform the COVID-19 detection and quarantine at the local centralized isolation points for 14 days. The transportation system is currently strengthening its epidemic response measures to international travel, to prevent a second outbreak caused by imported cases.

## 3. Implications for Other Countries 

In the early stage of the epidemic, the outbreak mainly occurred in China, and many countries such as the US and Australia had implemented aggressive policies to restrict the entry of Chinese or non-native visitors. However, because of its pandemic nature and global distribution, there is currently no scientific evidence to confirm that COVID-19 originated in China. Since March 2020, the outbreak has gradually spread in more than one hundred and fifty countries, and the precious opportunity that China has won for the prevention and control of the epidemic is being expanded globally.

As a report by WHO notes, “China has rolled out perhaps the most ambitious, agile, and aggressive disease containment effort in history” [15]. Based on the current epidemiological data (see Figure 1), the Chinese government has effectively implemented multiple measures against the COVID-19 epidemic, showing a dramatic decline in new cases across China. These measures that have been put into practice in China helped “buy time” for the spread of epidemic, and other countries could consider taking lessons from China’s successful measures, and adjust strategies based on their own conditions and the new variation of the virus. 

For the countries with an outbreak of COVID-19, the highest-level emergency response plan should be launched immediately. All necessary interventions should be taken, such as limiting assembly, closing schools and public places, and implementing social quarantine, to restrict the direct spread of the epidemic in and across community. Cases should be quickly and timely checked and traced by using big data and new technologies, and confirmed cases, suspected cases, and close contacts should be strictly isolated. Medical emergency resources need to be supplied by all means. If necessary, a new batch of specialty field hospitals and mobile cabin hospitals should be constructed in order to treat the cases in different types and stages. The propaganda work of epidemic prevention and control must be strengthened to make residents fully aware of the importance of COVID-19. When going out, they are required to wear masks and keep social distancing.

For uninfected countries, they should be ready to launch the emergency response mechanisms at any time, and immediately strengthen the ability for a fast detection of the COVID-19, isolation of large-scale cases, and rapid supplementation of medical resources. Temperature monitoring and virus detection of foreign visitors should be strengthened at each customs port, in order to control the spread of epidemic caused by imported cases.

More importantly, although each country has its own special institutional environment, policy and culture, the global spread of the COVID-19 epidemic has no national boundaries. It is critical for countries to put aside differences to share information and cooperate to fight the immediate public health threat.
